# Building consensus for harm reduction approaches in UK universities: a qualitative study with staff and students

**DOI:** 10.1186/s12954-025-01351-4

**Published:** 2025-12-01

**Authors:** Karen Duke, Betsy Thom, Rachel Drayson, Camille Alexis-Garsee, Katie Anderson, Naomi Graham, Swetha Guruprasad

**Affiliations:** 1 Middlesex University, London, UK; 2 Students Organising for Sustainability UK, Middlesex University, London, UK

**Keywords:** Harm reduction, Universities, Students, Policy, Consensus, Stakeholders, Drugs, Co-production

## Abstract

**Background:**

Harm reduction approaches to student drug use have been developing in a number of UK universities. Little is known about how to build consensus and effect normative change towards harm reduction in these settings. The aim of this paper is to identify key factors relevant to achieving organisational change and effecting a shift towards the adoption of harm reduction policies and practices.

**Methods:**

The study included twelve UK universities and employed a qualitative research design which included semi-structured interviews with university policy makers (n = 12) and focus groups with students (4 groups with an overall total of 24 students). The universities were at different points in the process of implementing a harm reduction approach.

**Results:**

There was strong support for harm reduction approaches by both university staff and student participants, but there was confusion and misunderstanding around the concept of harm reduction. Some students were unsure about whether or not their university had a harm reduction policy. This highlights the need for clearer communication and education around harm reduction. A number of challenges in developing and implementing harm reduction strategies were identified including securing resources and gaining institutional support and leadership ‘buy-in’. Fears of stakeholders around the reputational risks to universities in advocating for harm reduction needed to be countered. Policy change often relied on committed individuals or ‘champions’ in the institutions to drive strategy development; however, there was also evidence of a ‘community of practice’ emerging amongst the network of institutions that had implemented harm reduction strategies which provided support and shared knowledge and experiences. Co-production strategies involving students, staff and external stakeholders were vital in gaining consensus and fostering ownership.

**Conclusions:**

The study has highlighted that there is strong support for a shift away from zero-tolerance approaches to student drug use, although this needs to be accomplished incrementally and in ways that address stakeholder concerns and safeguard university reputations. Working with a ‘whole system’ philosophy was identified as good practice whereby university policy was embedded within a local or city-wide approach which helped to facilitate the acceptance and development of harm reduction.

**Supplementary Information:**

The online version contains supplementary material available at 10.1186/s12954-025-01351-4.

## Introduction

In 2019, an independent review commissioned by the UK government, detailed the challenges posed by current drug use and drug-related harms and set the framework for a national strategy for prevention, treatment and recovery [1–3]. The Black Reports highlighted young adults as an important target group for prevention [1, 2]. It was noted that drug use peaked in the 20–24 age group for Class A and all drugs, and among those age 16–24 (an age where many young people are still in educational institutions), prevalence rates in the last year were highest for cannabis use (17.3%), followed by nitrous oxide (8.7%), powder cocaine (6.2%) and ecstasy (4.7%). Males were more likely to report use of all substances compared to females. Students were only slightly less likely to use drugs compared to the total population aged 16–24, but the evidence pointed to the use of ecstasy and nitrous oxide being higher among students than among non-students of the same age ([1], p. 72–74). More recent figures from the British Crime Survey (2024) indicate little change in drug use for people aged 16–24 years, although there has been a slight decrease in any drug use since 2020 [4]. In the year ending March 2024, prevalence rates in the last year for those aged 16–24 years were highest for cannabis use (13.8%), followed by powder cocaine (3.8%), nitrous oxide (3.3%), and ecstasy (2.2%). Around 150,000 young people aged 16 to 24 years (2.5%) were frequent drug users (took any drug more than once a month) in year ending March 2024.

Moyle and Coomber have described the university as a ‘risk environment’ which can provide the right structural conditions to facilitate a shift from drug use into drug supply, e.g. through freedom, opportunities, relationships and cultural environments that enable offending [5]. Reviews of the literature indicate that very little is known about the extent or detailed characteristics of drug use, drug supply and drug-related harms in the UK student population [6–8]. A review by Boden and Day showed that there is no coherent body of research providing a good picture of student drug use in the UK [7]. The authors found that, since the 1980s, prevalence of reported drug use had crept up, the range of substances reported had broadened and attitudes to drugs had, in general, normalised [9]. This review did not systematically assess the quality of the included studies, but did note significant methodological issues, such as small, narrow sample sizes and no distinction between lifetime/regular use, that limit the utility of the findings. Moreover, there was very little evidence of published work on prevention or on interventions to reduce drug-related harms [8].

Another review of 50 papers from the UK and Ireland, published between 2011 and 2021, supported Boden and Day’s findings and noted a lack of data on drug use prevalence among people from different ethnic and racial backgrounds and data on people with protected characteristics [10]. One UK study pointed to women, LGBTQ+ and disabled students as being significantly more likely to name motivations for taking drugs linked to their mental health and noted that disabled students were more likely to have used drugs to self-medicate for a physical health problem [11]. International research (largely from the USA) also provides evidence that LGBTQ+ students may be more at risk of harmful drug use [12–15]. Findings from international research also indicate that discrimination associated with ethnic minority status is a risk factor for negative coping behaviours such as drug use [16–19] and that students attending universities outside their home countries are more likely to use drugs to cope with stresses arising from the need to adapt to a different culture, language and social status [20]. These studies highlight the importance of addressing drug use and drug-related harms among specific student groups who may be more at risk of harmful drug use and examining the development of policies and interventions which reduce harm in university settings.

Student wellbeing is widely recognised as a priority in higher education, particularly in relation to mental health and suicide [21] and the impact of discrimination is well documented [22]. However, there is little open discussion of illicit drug use and associated harms. To fill this gap, Universities UK (UUK), in partnership with Unite Students, set up a Drugs Taskforce in 2022 to compile a better evidence base for understanding and responding to university students’ drug use and to provide practical recommendations for action. A literature review and new representative survey (n = 3989 students from across the UK) carried out by Savanta ComRes, found that around 18% of students had ever taken an illicit drug and within this group, two-thirds had used drugs in the previous 12 months, equating to 12% of those surveyed [8]. This figure is notably lower that the rate of past year use for 16–24 year olds (16.5%) in England and Wales [4]. Of those who had ever taken drugs, the most commonly used drug in the last twelve months was cannabis (53%) followed by cocaine (8%), prescription drugs (7%), ketamine (6%) and ecstasy (4%) [8]. Nearly half (44%) of the students who had used drugs in the past 12 months reported wanting to reduce their drug use, but they reported a range of barriers to help-seeking for drug-related issues; 54% said that stigma was a barrier, and 46% cited concerns about their university’s policy on drugs [8]. Importantly, concerns were raised by the UUK student survey that zero-tolerance approaches to drug use acted as a disincentive to help-seeking for those students needing support for drug-related harms. There was also evidence from the wider literature that adopting a zero-tolerance approach did not reduce the prevalence of use, but did have an adverse effect on student welfare [8, 11, 23]. Student concerns about the possible repercussions of revealing drug use and related problems was a key consideration driving the Taskforce in their examination of university policies and responses to drug use and an attempt to move policy in the direction of harm reduction approaches.

The UUK Framework for Action, *Enabling student health and success. Tackling supply and demand for drugs and improving harm reduction,* was published in July 2024 and concluded that ‘*evidence points to a need to give primary focus to reducing harm*’ ([8], p. 3). The framework states clearly that, ‘*A harm reduction approach does not involve condoning or seeking to normalise the use of drugs. Instead, it aims to minimise the harms which may occur if students take drugs*’ ([8], p 8). The report also points out that many universities already take a nuanced approach in responding to student drug use combining both deterrent and supportive approaches and taking account of the specific circumstances surrounding drug use and drug supply incidents. By June 2025, an analysis of Freedom of Information requests to 144 UK universities to assess their adoption and implementation of the UUK report’s recommendations showed that one third (34%) had adopted harm reduction policies; one-third (33%) were developing policies; and a significant minority (13%) had maintained zero tolerance approaches [41].

By the time the UUK Framework for Action was published, work was already underway to promote a harm reduction approach in universities. Recognising the need to address issues such as consensus building and effecting normative change towards harm reduction as a way of responding to drug use, Students Organising for Sustainability UK (SOS-UK) launched ‘Drug and Alcohol Impact’ (DAI) in 2020, a programme aiming to support universities to develop drug harm reduction policies and interventions to reduce the negative outcomes of student substance use. The SOS-UK DAI programme outlines the key characteristics of a harm reduction approach which places the health, safety and well-being of both students who may use drugs, students who do not and others such as members of staff and local communities at the centre of any response. Harm reduction does not condone or normalise the use of drugs, but aims to minimise the wide range of harms which may result from drug use and supply. Universities adopting a harm reduction approach work in partnership with the health system, other support services and local police forces. Their approach may involve efforts to inform about the risk of drug use, practical interventions to improve safety and campaigns to reduce stigma and encourage access to support. A harm reduction approach does not necessarily exclude the possibility of disciplinary action being taken against students (particularly where there is supply or where use causes harm to others). However, disciplinary action is not at the foreground of harm reduction policies relating to drug use.

By summer 2025, the DAI programme had worked with 16 UK universities to develop harm reduction policies. This work includes: (1) reformed disciplinary approaches (i.e. adopting the use of warnings, use of educational conversations, provision of voluntary or mandatory engagement with support, and no further action outcomes AND reducing use of fines, disciplinary processes being initiated after one incident, and not mentioning support in policies); (2) rewritten policy to make it more student-friendly and accessible, including communication about the policy change; (3) an explanation of what harm reduction means to them and their students in the form of a commitment statement; (4) reference to other policies that may interact with substance use (e.g. disciplinary policies and residential agreements); and (5) clear review dates to ensure policies are kept up to date with best practice.

In collaboration with SOS-UK, Middlesex University Drug and Alcohol Research Centre (DARC) secured funding from the internal Higher Education Innovation Fund in 2024 for a project to synthesise the knowledge gained so far from SOS-UK efforts to introduce harm reduction policies and approaches in UK universities. Given the lack of research on developing substance use policies in universities, the aim of this project was to identify key factors relevant to achieving organisational change and effecting a shift towards the adoption of harm reduction policies and practices in response to student drug use. To do this, the research team spoke with students and staff at universities that had already adopted, or were in the process of adopting, a harm reduction approach to student drug use. The project objectives were:

- to explore students’ and staff understandings of ‘harm reduction’ as an approach, their ideas about whether a harm reduction approach was acceptable/ practical, and their experience of university-based policies and interventions.
- to understand barriers/ facilitators of (a) getting harm reduction accepted as an approach, (b) getting it implemented into university policy/ practice.
- to collect information on *how* people have gone about securing normative change – what approaches/ strategies they used, and which proved successful in what circumstances.
- to understand the impact of wider community contexts (and stakeholders) on universities’ drug policy and practices and on securing a shift to a harm reduction approach, with the aim of working towards a more comprehensive, inclusive approach that recognises universities within their situational and environmental contexts.

This paper reports on student and staff understanding of harm reduction as an appropriate framework for responding to student drug use, on perceived barriers and facilitators to securing a shift in university policy and practice away from zero tolerance towards harm reduction, and on the strategies adopted to promote change in university policies and responses. Although the DAI project includes alcohol and drugs, study participants stated that alcohol was far less controversial than drugs with respect to implementing harm reduction policy; for that reason, this paper concentrates on findings regarding student use of illicit drugs.

In the next section, we outline the research design and methodology. This is followed by the findings from staff interviews and student focus groups where we report participants’ perspectives on harm reduction, the challenges to adopting a harm reduction approach in universities, experiences of building consensus and implementing harm reduction policies and interventions in Higher Education. Finally, we discuss the need for a whole system approach to address the UK Government’s aim to reduce drug demand.

## Research design and methods

Although the project did not adopt any specific theoretical frame, it was informed by policy science perspectives [24] which influenced the questions posed to staff and students. We were interested in how our respondents retrospectively recounted organisational policy change from initial efforts to influence policy agendas through to examining the process by which policy change occurred (or not), the factors that influenced the process, and the role of different individuals, networks and ‘change agents’ in effecting change.

The study included twelve universities located in the UK and employed a qualitative research design which included semi-structured interviews with key informants (n = 12) and focus groups with students (4 groups with an overall total of 24 students). Qualitative methods were most suitable for data collection as we aimed to understand the perspectives and experiences of those involved in the process.

### Interviews

The key informants were staff members in senior positions in university professional services. They were selected for the study due to their strategic positions in relation to the development of substance use policy in their universities. Interviews were conducted with nine key informants from nine universities which had adopted the DAI programme designed by SOS-UK. This programme is underpinned by a harm reduction ethos. These universities were at different points in the process of implementing a harm reduction approach and becoming accredited under the DAI programme. The aims of the interviews were to understand the barriers and facilitators of getting harm reduction accepted as an approach and implemented into policy and practice and to explore how key actors in the universities had gone about securing normative change (i.e. the interview schedules included questions on the approaches and strategies they used, and which proved successful in what circumstances, see Additional File 1 for the key informant interview schedule). Interviews were also conducted with three key informants from three universities which had considered, but not taken up, the DAI programme. The interviews with key informants lasted approximately one hour and were conducted by the researchers at the Drug and Alcohol Research Centre at Middlesex University. They were recorded and transcribed via Microsoft Teams.

### Focus groups

Two focus groups were conducted with students attending the universities with the DAI programme (n = 8; n = 5) and two focus groups with students from other universities which had not adopted the DAI programme (n = 7; n = 4). Students were recruited through the TOTUM student discount cardholder database [25] and through direct communications from the DAI partnerships where this was possible. A screening survey was used for students to register their interest, indicate their availability and provide demographic information (i.e. age, gender, year of study, and domicile). Of the 24 students who participated, 9 had ever used an illicit drug and 17 had ever used alcohol. To allow for a good mix of students in relation to demographic characteristics and to capture the range of perspectives within the student population, the information provided was used to identify which students would be invited to participate (See Table 1).

**Table 1:** Student focus group participant demographics

		DAI Universities Groups (n = 13 participants overall)	Non-DAI Universities Groups (n = 11 participants overall)
Domicile	UK Students	7	9
	Non-UK Students	3	2
	Not given	3	0
Gender identity	Men	2	6
	Women	8	4
	Non-binary	1	1
	Not given	2	0
Age	18–29 years	10	6
	30+ years	1	5
	Not given	2	0
Year of study	1st Year	2	0
	2nd Year	3	4
	Final Year	1	3
	Not given	7	4

The focus groups were convened by SOS-UK researchers on the Visions Live platform [26]. This is an online qualitative research platform designed to manage text-based focus groups and offers a closed, invite-only environment. This meant that participants from a broad geographical region joined together to share their views and allowed for assessment of experiences from different parts of the UK. The text-based nature of the interactions means that participants are likely to feel a greater degree of anonymity and therefore are potentially more likely to be open and honest in their discussions and answers, particularly in relation to sensitive topics like substance use policy [27]. It may also allow participants who may not feel confident speaking on camera in a group discussion to contribute. The aims of the focus groups were to examine students’ understandings of ‘harm reduction’ as an approach, their ideas about whether a harm reduction approach was acceptable/practical in the case of illegal drugs and to explore students’ experiences of university-based policies and interventions (see Additional File 2 for the student focus group schedule). The focus groups lasted one hour and involved the moderator posing questions via text-chat and running activities via an interactive whiteboard within the platform. Throughout the synchronous text-based discussion, participants responded (via text) directly to the moderator, or to points made by other participants.

### Ethics

The project received ethical approval from the Law and Social Sciences Ethics Committee at Middlesex University (ID27723). The participant’s data was anonymised by using identity numbers and pseudonyms for individuals/organisations taking part in the interviews and focus groups. The university names have been given pseudonyms in the text (e.g. DAI Uni 1 and non-DAI Uni 1) to ensure that they cannot be identified. Key informants were sent a participant information sheet prior to the interview and written informed consent was gained. All students were emailed the participant information sheet and consent form prior to the focus groups. The research project was introduced and informed consent was obtained through the text-based chat at the beginning of the session. The students were given £20 to compensate for their time.

### Analysis

The data generated from the interviews and focus groups were analysed thematically according to Braun and Clarke’s approach [28, 29]. Our inductive thematic analysis involved reading through the transcriptions from the interviews and focus groups, generating initial codes on these documents, bringing the codes together to search for themes and defining the key themes. The research team conducted a preliminary thematic analysis on a small number of transcripts, which were discussed in a team meeting and used to develop a provisional coding framework of ten themes. After further analysis, the team met again to expand and refine themes in the coding framework, before applying the framework to the remainder of the transcripts. This resulted in four key themes (see Table 2 below with themes and sub-themes). Initially, we conducted separate analyses for the staff interviews and student focus groups and then brought them together. The main focus of this paper is on building consensus for a harm reduction approach and the reflections of the staff participants on how they went about this in their institutions. Staff perspectives are therefore more prominent in this particular paper. Where data from the student focus groups aligned to the themes generated from the staff interviews, we have included these.

**Table 2:** Themes and sub-themes

Themes	Sub-themes
Defining/understanding harm reduction	Staff: identification of key characteristics of harm reduction
	Student: mixed understandings of harm reduction
	Student awareness of university drug policy
Challenges to harm reduction	Reputational risk and stigma
	Resources and priorities
	Getting stakeholders on board
Building consensus	Personal interest & investment of initiator(s)
	Co-production with students
	Communication
Doing harm reduction in practice	Securing stakeholder buy-in
	Drafting and reviewing the strategy
	Knowledge exchange and diffusion of learning
	Incremental change towards harm reduction

It should be noted that two of the authors (RD and SG) are employed by Students Organising for Sustainability (SOS-UK) in their Research Team and the Drug and Alcohol Impact (DAI) Programme is delivered by SOS-UK. The remaining authors (KD, BT, CA-G, KA, NG) are all employed as academics at Middlesex University, but do not have any involvement in the development of substance use policies at the University. To try to minimise bias in the data collection, analysis and interpretation of the data, the University researchers undertook all the interviews with university staff participants, while the SOS-UK researchers led the focus groups with student participants with Middlesex research team members also present. All researchers were involved in the analyses and interpretation.

## Results

Findings from the interviews with university staff and the focus groups with students are presented under the 4 key themes identified in the analysis.

### Defining and understanding harm reduction

#### Staff: identification of key characteristics of harm reduction

All the staff participants from both DAI and non-DAI universities expressed strong support for a harm reduction approach which they saw as appropriate and acceptable in the university context. However, it was clear from the interviews that they struggled to provide a clear definition of what they meant by the term ‘harm reduction’, but they were able to discuss the key characteristics of a harm reduction approach. This was described as keeping students safe and reducing harm from substance use. This included both students who use substances as well as the people around them.

*I don't know if we define what harm reduction is...What we do say is that we recognise that the most appropriate approach to drug use is education and support with a primary aim of keeping people safe* (DAI Uni 9 Staff).

*…reducing the harm to yourself by the misuse of alcohol or drugs... misuse doesn't mean not using at all…It's about not putting yourself in danger by your use. And it's about reducing harm to the people around you* (DAI Uni 7 Staff).

The staff participants pointed to the failure of zero-tolerance policies, but they stressed that they were not condoning drug use. A key dilemma was striking a balance between tolerance and risk management.

*We know that zero tolerance has been failing…It leaves people isolated and stuck with an issue feeling unable to come forward. [A harm reduction approach] isn't condoning drug use. This isn't saying that we're going to accept any behaviours around this, but we need to have a space where people feel comfortable to come forward and share their experiences* (DAI Uni 1 Staff).

The harm reduction approach was characterised by raising awareness, providing education and support through open and non-judgemental conversations so that students could make informed choices about their substance use.

*Harm reduction is about opening up conversations around substance use; what's dangerous, what's not, what might be in the local area that students need to know about, what they may not be aware of; clear non-political messages to help them make well informed choices about their own behaviour* (Non-DAI Uni 2 Staff).

Staff recognised that it was more difficult to gain acceptance of harm reduction with respect to students’ drug use compared to alcohol use. This stemmed from the illegal status of drugs which made discussions on drug use ‘*a bit more taboo’* (DAI Uni 3 Staff), aroused ‘*fear and judgement and all that kind of thing*’ (Non-DAI Uni 2 Staff), and because alcohol is ‘*less stigmatised’* than illegal substances (DAI Uni 3 Staff).

#### Students: mixed understandings of harm reduction

Like the staff, student discussion in the focus groups revealed some confusion surrounding the concept of harm reduction. They tended to see harm reduction in ‘prevention’ terms whereby the goal is to stop or prevent further use, rather than managing the risks associated with continued use. One student described harm reduction as:

*Giving support to avoid a problem getting any worse and trying to stabilise at least or best reduce/stop further use (Non-DAI Student Focus Group 1, Participant 1).*

Some students were unaware of the term prior to participating in the group; *“I’ve not heard the phrase before, but I would think preventing escalation, early steps” (Non-DAI Student Focus Group 1, Participant 4)* indicating that the term ‘harm reduction’ itself is not widely understood. This lack of familiarity with the concept may contribute to the confusion between harm reduction and prevention.

While there was agreement on the need for harm reduction, students’ understanding was varied and conflicted. Some saw harm reduction as involving education about drug use and providing safer use environments (e.g. drug checking facilities), focusing on public health interventions, while others leaned towards more restrictive approaches, including reduced access and a zero-tolerance stance. Despite mixed understanding of the concept of ‘harm reduction’, participants acknowledged the importance of providing a supportive and educational approach for managing drug use at universities:

*Support, education and an amnesty-style approach that encourages students to discuss/disclose if there is concern for safety or other criminal activities etc - the less punitive, the better (DAI Student Focus Group 1, Participant 6).*

They acknowledged that drug use, particularly among university students, is prevalent and unlikely to be eradicated, making it critical to provide safe and supportive environments.

*People are always gonna do drugs at university, so it's important to make sure that they're aware of how to safely do so (DAI Student Focus Group 1, Participant 7).*

#### Student awareness of university drug policy

Confusion and uncertainty about the concept of harm reduction was reflected in students’ comments on their knowledge and experience of university drug policies. Several students expressed uncertainty about the existence of a formal policy at their university. Among those who were aware of such policies, many admitted to not fully understanding the details, due to them being unclear or not well communicated. For instance, some students recalled being informed about a ‘zero tolerance’ drug policy upon joining the university but noted that this message was not reinforced throughout the year.

*I know that there is both a drugs and alcohol policy at my university as I have received communication about this via email...however, I don't know these policies in-depth, they are quite long documents that are pretty wordy! (Non-DAI Student Focus Group 2, Participant 9)*

In some cases, student participants expressed uncertainty about whether their university’s approach was supportive or punitive. This often stemmed from conflicting messages or inconsistencies between different branches of the university, such as between the university administration and the SU or between university policies and student accommodation policies. One student pointed out that while their university outwardly promotes a harm reduction approach, the actual policies in place may reflect a more punitive, zero-tolerance stance:

*My uni is supposed to be harm reduction but I’m pretty sure the policy I got at the beginning of my degree was far more of the zero-tolerance (drugs) vibe (DAI Student Focus Group 1, Participant 7).*

Another student mentioned the discrepancy between university and accommodation policies, where the latter might be more punitive:

*Supportive when it comes to the university in general, however accommodation policies can be more punitive (DAI Student Focus Group 1, Participant 2).*

There was a perception that students’ union and university policies did not fully align:

*Harm reduction is clearly part of the Student Union ethos...but I don't think the university would associate itself with harm reduction ethos (Non-DAI Student Focus Group 2, Participant 9).*

Ensuring that staff and students understood what a harm reduction approach meant and communicating clearly how harm reduction was applied in university policies and practices emerged as one of the key challenges in initiating organisational change away from a zero-tolerance approach. Both staff and students identified several other challenges to initiating and embedding a harm reduction approach as discussed in the following section.

### Challenges to harm reduction

Challenges included reputational risk—how harm reduction was perceived by students, their parents and the media; lack of available resources and prioritisation; and the difficulties associated with getting and keeping stakeholders on board.

#### Reputational risk and stigma

Many university staff spoke about how a harm reduction approach might be perceived by students, parents and wider society. They voiced considerable concern that openly advocating for harm reduction might damage the university’s reputation, citing the stigma associated with illicit drug use and certain harm reduction practices. The fear of reputational risk was made even more pressing by the current climate, where universities are facing financial issues and were focusing on boosting recruitment. Adopting a harm reduction approach could give the impression that drug use was a systemic problem within the university. This concern was intertwined with the potential for harm reduction to be misunderstood as condoning drug use, whereas ‘zero tolerance’ was seen as clear-cut and hard to misinterpret. University senior leaders had sometimes expressed concern about how a harm reduction approach would look to the public, and, in particular, to the media:

*I talk to either a senior leader in the university or a professor and they'll be saying, ‘what, we're giving out drug testing kits? How's that going to look in the Daily Telegraph or Daily Mail or whatever?’ So that reputational aspect does bring a practical challenge* (DAI Uni 2 Staff).

Student participants largely agreed with staff concerns, noting that public perceptions, could be damaging. One student stated, *"reputation at risk depending on how involved universities get” (DAI student Focus Group 1, Participant 8).* Another student expanded on the reasons why harm reduction could be perceived negatively:

*Local communities close to universities, people who've not experienced drug or alcohol problems, won't understand the need to receive support rather than punishment (Non-DAI Student Focus Group 1, Participant 2).*

#### Resources and priorities

Both staff and student participants pointed out that substance use policy was not given a high priority in relation to other institutional concerns. It was hard to make a case for harm reduction as the highest priority, especially when other issues might impact students more, like mental health, or issues such as freedom of speech and sexual misconduct.

*There are a huge amount of competing priorities…This would not be at the top of the list, because there's things like freedom of speech, which we've got a legislative and regulatory requirement to do properly…harassment and sexual misconduct, which will also be a regulatory requirement. So those things have to take priority over anything else…there are budgetary issues*…(Non-DAI Uni 2 Staff).

The time and resources needed to adopt a harm reduction approach presented a significant challenge as staff time and resources were both in short supply.

*Most institutions now are very stretched in terms of their resource space, you've got staff already very busy in roles…[but]…everyone's worked incredibly hard* (DAI Uni 5 Staff).

Some universities were able to work through this resourcing challenge, whereas for others a lack of *‘the time and energy…this piece of work needs’* (Non-DAI Uni 2 Staff) represented a barrier to even getting started. In the student focus groups, logistical challenges like cost, space, and institutional support were frequently cited as barriers to the rollout of a comprehensive harm reduction approach.

*Probably not wholly possible for many reasons—cost, time, space, support (Non-DAI Student Focus Group 1, Participant 1).*

#### Getting stakeholders on board

Another challenge related to getting stakeholders (both internal and external) on board to support the harm reduction approach. Within universities, even when support for a harm reduction policy had been secured, staff turnover could threaten to stall or complicate progress, so commitment had to be renegotiated. This was flagged with respect to the Student Union (SU), where union officers changed year-on-year and there was no guarantee newly elected officers would be supportive. At one university, cultural differences meant new SU officers from different ethnic and religious backgrounds had more punitive views on drug use, which cooled the enthusiasm of their support for harm reduction policies. For another university, there was concern that unsympathetic SU officers could become a total barrier in future:

*There's this never ending churn of sabbatical officers...The current [officers] all buy in, but who knows if the next [officers] will…a president who didn't agree with it…that would cause us problems because we couldn't overturn that election* (DAI Uni 7 Staff).

Conversely, a changeover in staff could also work in favour of a harm reduction approach as previous staff who had been very resistant moved on and harm reduction could be presented as the status quo to new staff who were more receptive:

*There's been a change in [security] manager and [they’re] much more open to having this discussion…historically, security was a barrier, but a change in staff and us being there ready to introduce ourselves and go, ‘hey, this is what the university does’. They were able to get more on board.* (DAI Uni 1 Staff).

Reaching out to community stakeholders also presented challenges. It could be difficult to identify who the relevant stakeholders were and then difficult to convince them of the merits of harm reduction:

*Just getting your stakeholders on board is probably one of the biggest things...you've got to have community stakeholders on board as well. It's not just internal and…that can be really tricky* (DAI Uni 3 Staff).

*It can be difficult to navigate some of the larger institutional groups…especially with the NHS, it's complex…finding the right colleague at the right level* (DAI Uni 2 Staff).

Several stakeholders were highlighted by staff participants as more difficult to engage, such as senior leadership, police, security, accommodation, and parents. Senior leaders were seen to be concerned for the university’s reputation if the institution became known as ‘accepting’ drug use, as discussed above. Whereas for the police and university security and accommodation services, there was a perceived clash between their legal obligations – as an enforcer of the law or a landlord – and the new policy. Families of students were also viewed by staff participants as a possible cause for concern, as parents wanted universities to discourage drug use. However, the extent to which stakeholder groups were comfortable with harm reduction varied within and between institutions. For one institution, the police were viewed as the most difficult stakeholder to bring on board, whereas for another university, the police were seen to be strong advocates for and enablers of a harm reduction approach.

Addressing these challenges required considerable effort on the part of staff members leading the harm reduction policy to counter the fears and misconceptions of the different stakeholder groups as well as building consensus around the value of a harm reduction approach, fostering and embedding appropriate support and commitment to change.

### Building consensus

The staff participants identified several facilitators which helped to build consensus around a harm reduction approach. This included having a personally invested initiator and engaging students using a co-production approach. They also stressed the value of drawing on the existing evidence to construct their case and the importance of communication, having an open dialogue with stakeholders and making communications digestible and accessible.

#### Personal interest and investment of initiator(s)

A key factor in building consensus was having someone with a personal interest and passion for harm reduction to help to drive forward the development and implementation of the policy and ensure that actions get completed.

*The only reason you and I are sat here today is because I am personally really interested in this area of work... There is actually nothing in my job description that says I should be doing this piece of work. It's not within my priorities* (Non-DAI Uni 2 Staff).

Without having a person with such an interest, staff participants felt that University committees, though useful, limited the work that was needed to get the job done, and this was echoed by another institution that used a ‘policy and operations’ group to engender change:

*That committee structure has its place, but it needs to be with the people that are going to ‘do the doing’…but no one's actually ‘doing the doing’. So, it doesn't actually get done* (DAI Uni 6 Staff).

#### Co-production with students

Staff participants stressed the importance of collaborating with students to co-produce the design and implementation of their institution’s harm reduction policy. They mentioned various strategies for involving students. For example, they stressed the importance of a bottom-up, student led approach rather than top-down decision making from senior managers within the university. This allowed students to take ownership of the initiatives and to embed these into student union policies and practices. In many of the institutions studied, the student unions played pivotal roles in raising harm reduction as the goal of policy and driving forward its development.

*I don't think we got the Union on board. They got us on board! And we agreed. That collaboration has got to be genuine…They are lobbying us to do it. Then we work together with them to achieve that. So that's quite positive* (DAI Uni 2 Staff).

In other institutions, the student union was not active and this was seen to hinder the development and implementation of harm reduction.

*We don't have a very active students union that is able to bring stuff like that together and that's for a number of reasons. So, how do you fill that gap?* (DAI Uni 6 Staff).

The inclusion of student voices in the development of communications and messaging was seen as vital in increasing understanding and legitimacy in the wider student body. Some of the institutions were developing specific peer roles for students and planned to train them in safety and harm reduction initiatives.

*The Students’ Union really did a great job at making it understandable to students and making sure they were aware of what is the key information that they need to know* (DAI Uni 3 Staff).

The importance placed on collaboration and co-production with students by staff participants aligned closely with the findings from student focus groups. The students called for policies that directly involve them in development and implementation so that they would be closely tailored to their needs. They suggested active debates, forums, and expert-led discussions to ensure that harm reduction strategies are well-informed and considerate of diverse student needs. As one student proposed,

*I would want it to be implemented with lots of student direct involvement, active debates and forums for views to be discussed (Non-DAI Student Focus Group 2, Participant 8)*.

#### Communication

Staff participants were attuned to the importance of communication around harm reduction and ‘getting the messaging right’ for all stakeholders, including students and parents. They highlighted the importance of ensuring the conversations they were having with key stakeholders were rooted in the evidence. Student surveys were key as these helped to tailor and target the harm reduction approach to emerging trends in student substance use, but also case studies could be used to illustrate alternative actions:

*We took some case studies about some of the more serious cases that we were dealing with and highlighted how this type of approach could help alleviate some of those issues that could have been identified earlier…some of our senior stakeholders weren’t actually aware of the things that we were dealing with…it really helped us make the case for harm reduction* (DAI Uni 3 Staff).

Open and honest dialogue was fundamental, and staff felt that all stakeholders should have the opportunity to interrogate the policy.

*You can get things wrong through your communications and marketing. It can have quite a lot of risk attached to it, because if you get that message wrong, it's quite difficult to rebuild that trust with people* (Non-DAI Uni 3 Staff).

*It's not like a secret policy. We've done a lot of student comms around the drug and alcohol policy. What does the harm reduction policy mean? While they're aimed at students, they're exposed to everybody at the university…It’s so important to give people that opportunity to share their concerns, their biases, and have that appropriately challenged* (DAI Uni 1 Staff).

Some institutions held roadshows to ensure that every member of staff and all students were able to learn about the policy and ask questions. Consistent and repeated communications were also important, so that each new intake of students is fully aware of the harm reduction approach. The communications for students needed to be visible, accessible, transparent and easily digestible. This involved communicating across multiple channels and building harm reduction messages within other campaigns so that this messaging became part of the University cycle:

*It's continuing with those messages and remembering that each year we get a new wave of people. This is a continuous thing that each year, with a new intake, we need to be re-installing these messages* (DAI Uni 3 Staff).

Making sure that relevant groups within the university were closely involved in the change process and had a sense of ownership over the development of policies and responses to drug use was clearly recognised by staff participants as fundamental to gaining consensus across the organisation. Similarly, taking account of external stakeholder concerns and the specific contexts in which they worked were crucial in gaining their engagement and support for implementing a harm reduction response within the wider community.

### Doing harm reduction in practice

Apart from promoting awareness and gaining consensus to move towards a harm reduction approach, there were a number of key components that participants viewed as important in enabling them to develop and implement harm reduction in their institutions at different levels. The ways in which they achieved this varied between the institutions, but common issues were raised around securing senior leadership support and stakeholder buy-in both within the university and with community stakeholders, fostering knowledge exchange and learning from other universities, and making sure the drugs policy and strategy were reviewed, up-to-date based on evidence. The staff participants also pointed to the importance of exchanging knowledge and learning from other universities to form a community of practice and mentioned the key roles played by SOS-UK and UUK in providing a framework for their work.

#### Securing stakeholder buy-in

Staff participants discussed the importance of university senior leadership buy-in, particularly at the highest levels (e.g. Vice Chancellors and executive level teams) and securing this right at the beginning of the process, in order to smooth the way for the acceptance of harm reduction as the guiding ethos of policy development and implementation, and to ensure the provision of resources:

*Number one is senior buy-in, senior buy-in at the start, not as an afterthought. Get it at the start. Don’t wait to put papers in. Go and talk. That helps. We’ve got a very open VC, you can go and talk to [them]. You need to talk to the DVC and people at the top early on. Buy-in – everything else is not easy, but it falls into place* (DAI Uni 7 Staff).

At the time of the interviews, the UUK guidance framework^8^ had not been published. Participants in the non-DAI institutions were waiting for its release as they believed this would help facilitate a shared understanding and commitment to harm reduction across the sector making it easier to implement in their institutions.

*Tell me when the UUK guidance is coming…have the conversation on the back of that guidance, because if not, it feels like it's me as an individual raising the conversation, asking the questions, whereas it's much easier to do that with senior leaders if we've got a sector move. If there is support for this across the sector and other universities are also doing it. So that's been our delay in asking those questions and getting that senior leader support* (Non-DAI Uni 2 Staff).

Effective implementation was also dependent on collaboration with both ‘the right’ internal stakeholders in each institution and with external stakeholders appropriate for their local areas. This could be challenging as there were so many different stakeholders that needed to understand the harm reduction approach, what is going on in different areas of the university and to ‘be on the same page’.

The plethora of internal stakeholders mentioned by staff participants as important to involve in the development and implementation of the strategy included the university executive team, the range of student support services (i.e. accommodation, well-being, finance etc.), campus security, catering staff, academic staff from all Faculties, PR and marketing, communications teams, legal services, student and staff unions, and students’ clubs and societies. This was seen to facilitate the development of a ‘whole university approach’ with ‘full university buy-in’. One of the participants referred to the benefits of including a cross-section of staff across the university involved in the steering group to help understand where the friction or problems around harm reduction might occur:

*It's really important to get a real cross-section across the university so that it is then a whole university approach and we can see what the pressure points might be…Having all of those bodies on the steering party is really important. We can then be solution-focused into what are we going to do about it…If everyone's cited on it from the outset and we can all be present and attend and discuss it as a party, we can then make sure we find the solutions to it* (Non-DAI Uni 3 Staff).

The PR and marketing team was viewed as crucial to the development, implementation and communication of the policy. Many of the institutions struggled with getting their PR/marketing team to buy-in to the approach and they needed to spend time convincing them to agree to put out communications around the strategy:

*It’s the marketing team that were the biggest challenge, just even putting out messages about this policy on social media. They really struggled with it. We had to do a bit of bartering around that. But eventually it’s just become so normal* (DAI Uni 3 Staff).

One way to stimulate buy in was to ensure that staff and students understood what harm reduction meant and what it entailed in practice. Universities had implemented harm reduction training in a variety of ways to build knowledge and understanding of harm reduction, develop the practical skills needed to ‘do’ the approach and to raise engagement and awareness. This was an important pre-cursor to the implementation of the strategy. Providing student support was a common thread throughout the training. As one staff participant stated:

*So, the first approach is primarily support. So, we have training for staff to notice if a student is in need, check that they've understood it correctly. And then with that student's consent if there's a risk, to share that with us, if there is risk staff know that they can contact us* (DAI Uni 9 Staff).

External stakeholders in the community were also important to involve at all stages of the development and implementation of the strategy. Often, this took much negotiation and discussion to get them on board. One participant outlined how changes in the staffing of the external stakeholders and their priorities and requirements could threaten the implementation of the strategy going forward:

*The biggest threat is the local police, if they turn around and say we need to be made aware of every case, that completely defeats the point of our whole policy…I don’t see any internal threats to the policy. Everyone’s very supportive and it has been working. It will be external stakeholders. If, for example, our local recovery service said they can no longer support students, that would leave us in a bit of bind as to how we can refer students to external support…The changeover of staff and their different priorities and their different ways of working…they are the main things* (DAI Uni 3 Staff).

In the focus groups, the students widely supported the idea of involving external organisations, such as professional bodies and charities, in implementing harm reduction. Many students thought that such collaborations with external partners could enhance legitimacy and effectiveness.

#### Drafting and reviewing the strategy

The steering group was a key mechanism for drafting and developing the strategy in all the DAI institutions. Staff participants pointed to the importance of the various iterations of the document going out for comments multiple times in order to get the balance right between supply reduction, demand reduction and harm reduction and also to maintain buy-in from all the relevant stakeholders.

*It was our project manager that authored it. But that was based on the inputs from everybody in that group. So, we've got the police in that group. We've got the night- time economy, we've got the licencing authority. We've got academics and obviously student voice representatives. It was a whole mix of all of those colleagues contributing towards [the policy shift to HR]* (DAI Uni 8 Staff).

The wording of the strategy document was seen to be crucial, so that all stakeholders, including students, could easily understand the implications of the strategy and what would happen in practice. One of the universities was in the process of re-drafting their strategy and discussed the differences between the first version which was quite abstract and conceptual and the current version which was much more practical and student-friendly:

*We found that it didn’t feel particularly student friendly. So, the approach isn’t hugely different, but trying to make it really clear to students what it means for them…It’s a policy for students, not us. And much clearer on professional suitability, what that means for them and a bit more on pathways on procedures. So, people can pick up the document and go, ‘OK if I talk to this person, what can I expect to happen’. Because it felt more of a statement or a concept – the first policy we had. We wanted it to feel a lot more practical* (DAI Uni 1 Staff).

This emphasis on the practical aspects of the strategy was also discussed in the student focus groups. They expressed the need to make sure that the policies were clear as to what would happen in different circumstances. One student noted a lack of clarity regarding the specific consequences, which are often inferred from anecdotal evidence rather than official university communications:

*I think repeat offenses would result in expulsion, but police escalation is unlikely apart from possibly in the case of dealing: but again, I emphasise that this has never been spelled out fully by the university! I assume this is the case based on anecdotes from others and the attitude of the uni to student behaviour overall (Non-DAI Student Focus Group 2, Participant 9).*

However, there was widespread agreement in the student focus groups that harm reduction strategies should be applied on a case-by-case basis because not all substance use scenarios warrant the same approach. For example, in cases that involved violence, intent to supply drugs or harm to others, there might be a need for disciplinary responses.

The strategy needed to be seen as an agile, dynamic and live document which could be adapted depending on emerging issues. Regular reviews would ensure that it was fit for purpose. Tracking substance use in the student population through student surveys, understanding changing trends and identifying at risk and vulnerable groups were seen to be crucial activities in creating a responsive strategy and effectively targeting interventions.

*I’ll produce a report which will show the last three years or waves of data. So we can see where the trends are changing. And then we just try to be proactive in terms of recognising where the changes are and knowing that we’ll have to adapt our messaging. So, it’s just about targeting our messaging and making it as relevant as possible for our students* (DAI Uni 3 Staff).

#### Knowledge exchange and diffusion of learning

While much of the activity centred on securing internal and external support for the shift to a harm reduction approach and for the development of appropriate and acceptable policies and responses, those involved in the change process in universities and their surrounding communities benefitted from opportunities to exchange knowledge and experiences with other university networks. This helped them to feel supported and less isolated from other HE providers. In particular, staff participants pointed to the value of shared learning and knowledge exchange regarding best practice with other universities in the DAI programme. They were influenced by each other and borrowed from each other’s policy statements and strategies. It could be argued that they formed a ‘community of practice’.

SOS-UK facilitated this knowledge exchange and provided a framework and criteria that the universities could utilise as a basis for opening up discussions. They convened an annual conference which the DAI universities attended and played a pivotal role in inspiring institutions to adopt a harm reduction approach.

*We did take a lot of inspiration from the policies of other places. It’s great to be part of that group actually. It really helps to work through some issues that I think otherwise can feel like you’re in isolation with them* (DAI Uni 5 Staff).

Similarly, the UUK framework on student drug policy was seen to enable a sense of security within the HE sector around harm reduction approaches:

*Quite a few universities have gone down this track, so there’s a kind of security in that sense as sector we’re doing it and UUK were developing guidance around this. Sector wide – there is a recognition and that provides reassurance around some of the practicalities* (DAI Uni 2 Staff).

A few of the universities benefited from being embedded in a city or local area which promoted a city-wide or area-led harm reduction approach locally. The local universities were part of this wider partnership structure and strategy which enabled sharing knowledge, expertise and resources, and led to distributed responsibility for harm reduction and keeping students safe. For example, joint working and exchange of resources occurred between the night-time economy partners, police, drug treatment agencies, local taxi companies, and transport. The university did not have to take it all on alone or initiate all the ideas and practice around the harm reduction. As one participant explained:

*We are part of a harm reduction city. Our Council has embraced a harm reduction approach. This is a citywide approach…lots of other people have done a lot of the hard work for us* (DAI Uni 1 Staff)*.*

Nevertheless, despite the support afforded by belonging to a network of organisations dedicated to bringing about change, staff participants acknowledged that change needed to be incremental and that some measures were more controversial than others.

#### Incremental changes towards harm reduction

In universities where the DAI programme had not yet been commissioned or a harm reduction approach had not been adopted, there was still a commitment to implementing small changes towards harm reduction or embedding elements of harm reduction in other policies. As one staff participant pointed out, they were making small changes at the ground level towards harm reduction, without actually going all-in with an official policy commitment or document:

*We don’t currently have a policy which says we are working towards harm reduction in relation to student substance use. I don’t think that means there aren’t elements that we’re doing that would fit into a harm reduction kind of approach. It’s just that we’ve not enshrined that in policy* (Non-DAI Uni 2 Staff).

However, even in the universities where they had been working with an official harm reduction policy for some years, they suggested that with some measures (e.g. naloxone provision) which proved controversial in the university context, they were moving with caution, incrementally one step at a time:

*Naloxone is certainly on a future agenda. We’ve just had to go one step at a time in that cautious way. I’d like to get us doing general distribution…And it’s that kind of thing where you’re talking about small steps, incremental, working up to more controversial types of interventions* (DAI Uni 5 Staff).

For more controversial measures, it was recognised that the arguments around harm reduction would have to be re-iterated and re-negotiated with the university executive and placed on future agendas:

*One of the criteria is to get naloxone training, which wasn’t greatly approved, it was quite controversial. It will take some time to convince them, but in terms of the policy, everyone’s very aware that it does need to be changed and updated.* (DAI Uni 8 Staff).

These incremental changes towards harm reduction reflect what is happening across the sector one year after the publication of the UUK report [8]. Analyses shows uneven progress with two-thirds of universities moving towards harm reduction, but disparities and variations in implementation [41].

## Discussion

The findings contribute to the knowledge of what is needed to build consensus within universities, secure student, staff and external stakeholder awareness and acceptance of harm reduction approaches and establish appropriate policies and support systems.

There was strong support for harm reduction approaches to substance use in university settings by both staff and student participants. Harm reduction was viewed as both acceptable and practical.Nevertheless, there was confusion and misunderstanding of the concept of harm reduction, and students were unsure about whether their universities had a harm reduction policy or not. This highlights the need for clearer communication and education for staff and students about what the term ‘harm reduction’ means and what types of interventions it includes. Comments from staff and students also confirmed the need to counter stakeholder fears, both inside the university and among external stakeholders, that adoption of a harm reduction approach would damage the university’s reputation and affect recruitment.

Getting harm reduction on to university agendas at a high level posed problems. The staff and students had good awareness of the challenges of developing and implementing harm reduction strategies in their institutions including the problems of securing dedicated resources and gaining institutional support and ‘buy-in’ from leadership. Given the current pressures and economic crises in the HE sector, staff were aware of the problems around prioritising resources to this area of policy development. Nonetheless, they also had experience of what had helped to build consensus around the harm reduction approach.

As found in other studies of innovation, change often relied on ‘champions’ or ‘policy entrepreneurs’ [30, 31], committed individuals who initiated and drove forward the development and implementation of the policy or strategy. While the energy brought to the change process by such individuals can be key to its success, there are also risks, as other studies have documented [32]. Without a clear support structure to maintain change in the longer term, once such individuals move on, what has been achieved may fail to survive [32]. Given the prominent role of the SU in initiating and supporting innovation, and staff turnover in the universities, it may be difficult to embed and sustain change in that context. However, efforts to develop a network of universities implementing harm reduction policies and the emergence of a ‘community of practice’ [33] within this field, may go some way towards offsetting the risks of relying on short-term involvement of individual champions [31]. Through the DAI programme coordinated by SOS-UK, a community of practice has developed in the participating universities whereby they are not working in isolation, but sharing their experiences, exchanging knowledge and supporting each other in their harm reduction journeys. As Miech et al. note, implementation success is improved when multiple champions collaborate within their respective organisations and networks to lever change [34]. An additional safeguard against stakeholder and champion turnover comes in the form of using co-production [35] with students, staff and external stakeholders to develop and review policies and to gain consensus on appropriate responses to student drug use. Studies of partnership and joint working in health, education and other fields have highlighted the importance of fostering feelings of ownership as a means of securing ‘buy-in’, especially when working with stakeholders with different organisational goals and priorities, different professional regulations and practice methods, and different occupational cultures [36]. Co-production approaches have the potential, among other possible functions, to foster ownership through knowledge creation. As Bandola-Gill et al. report ([35], p. 285), research on co-production across different disciplines shares a common assumption that, ‘*knowledge required for addressing complex challenges is not produced solely within traditional structures but rather is developed across different communities and knowledge systems*’. Participant accounts of how they worked to gain collaboration between relevant stakeholders frequently mentioned co-production and consultation but did not indicate any clear strategic use of the approach. There is scope within the existing SOS-UK project to consider how current co-production approaches can be strengthened to expand stakeholder engagement and support policy development long-term.

As mentioned by some participants, working with a ‘whole system’ philosophy – which sees the university within the context of the wider community – was identified as good practice whereby university policy was embedded within a local or city-wide approach which helped to facilitate the acceptance and development of harm reduction. Evidence for using a city-wide partnership approach stems from insights derived from social systems theory which emphasises the importance of acknowledging the influence of ‘community’ and wider social/policy factors on securing change at institutional, organizational, and individual levels [37]. This theoretical approach has influenced research across different areas of health and education [38–40]. Currently, the SOS-UK project is focused on the university as the centre point for intervention. However, there is evidence of a move towards locating the university within the wider community context which was welcomed by some participants, and which encourages further steps in that direction.

## Conclusion

The findings from this research on SOS-UK’s DAI project are based on a small sample of university staff involved with the implementation of harm reduction policies, and with students who were not necessarily representative of student groups using drugs or most at risk of using drugs harmfully. We did not collect data from a wider range of university staff, including academic leaders or student union representatives, or from community stakeholders. Consequently, the findings may not represent the perceptions and experiences of other relevant individuals in the universities and communities. Despite these limitations, the study has highlighted that there is strong support for a shift away from zero-tolerance approaches to student drug use, although this needs to be accomplished incrementally and in a way that addresses stakeholder concerns and safeguards university reputations. Strategies adopted by the DAI projects demonstrate the importance of co-production and of developing strong partnership relationships with relevant stakeholders to gain ‘buy-in’ and engagement. We have suggested that the move towards a ‘whole system’ approach, by expanding the project into a city-wide collaboration between key organisations, enhances the chances of success and sustainability in the long-term as the original initiators and change ‘champions’ move on.

## Supplementary Information

Additional file 1.

Additional file 2.

## Data Availability

The datasets generated and/or analysed during the current study are not publicly available due to potential for identifying participants, but are available from the corresponding author on reasonable request.

## Abbreviations

SOS-UK: Students Organising for Sustainability UK
DAI: Drug and alcohol impact programme
UUK: Universities UK
HE: Higher education
‘Uni’: University
SU: Student union
