# Supplementary material for: Building consensus for harm reduction approaches in UK universities: a qualitative study with staff and students
Source: Harm Reduct J. 2025 Dec 1;22:195. doi: 10.1186/s12954-025-01351-4 (PMC12667090; doi:10.1186/s12954-025-01351-4)
Supplement: Supplementary file 1 — Additional file 1. [file 12954_2025_1351_MOESM1_ESM.docx]

**Building consensus for harm reduction approaches in UK universities: a qualitative study with staff and students**

**Interview Schedule for university staff (key informants)**

***Staff key informant interviews*** via Teams (staff in strategic positions in relation to drug policy in universities); includes universities at different points in the process of implementing harm reduction; to:

1. understand barriers/ facilitators of a) getting harm reduction accepted as an approach, b) getting it implemented into policy/ practice; and
2. collect information on *how* people have gone about securing normative change – what approaches/ strategies they used and which proved successful in what circumstances (etc.).

-----------------------------------

**Introductory Questions**:

1. What is your role in the university? What is your role in the development of the university drug policy/strategy/approach?
2. How long has your drug policy/strategy been in place? How often is it reviewed?

This first set of questions will be asked of key informants from institutions that have adopted a harm reduction approach and those that have not adopted a harm reduction approach.

1. **Understanding of Harm Reduction**:
2. Thinking about alcohol and drug use, what does the term ‘harm reduction’ mean to you?

Probe:

Is it different for alcohol and drugs?

**Definition of harm reduction**: *“Harm reduction refers to policies, programmes and practices that aim to minimise the negative health, social and legal impacts associated with drug use, drug policies and drug laws.*

*Harm reduction is grounded in justice and human rights. It focuses on positive change and on working with people without judgement, coercion, discrimination, or requiring that people stop using drugs as a precondition of support.”* (Harm Reduction International, 2024)

1. Can you provide examples of harm reduction approaches to a) drugs and b) alcohol? *For informants from universities that have adopted a harm reduction approach*, provide examples of harm reduction approaches to a) drugs and b) alcohol? Ask for examples.
2. In your view, is a harm reduction approach to drug use acceptable in universities? And is it acceptable for alcohol consumption? Probe reasons for answer.
   1. If yes, are there any circumstances where harm reduction *would not* be an acceptable approach?
   2. If no, are there any circumstances where harm reduction *would be* an acceptable approach?
3. In Universities, is it practical to adopt a harm reduction approach to a) drug use and b) alcohol use? Probe reasons for answer.

This set of questions will be asked for key informants from the universities which **have adopted** a harm reduction approach.

1. **Designing and Implementing a Harm Reduction Approach In Universities**

NB: They might take an approach but not have a policy in place. Unless it is specifically asking about the written policy, use approach. i.e. how has the "approach" been communicated within the university, rather than "policy"

1. Was your harm reduction approach initiated by a specific person or group in the university? When was the issue first raised? What prompted this? Does the approach/policy include alcohol. If not, are there two separate policies/approaches (one for illegal drugs and one for alcohol)? Why?
2. How did you go about developing your harm reduction approach? How did you secure consensus around harm reduction as the underlying principle of your approach/policy?

Prompts: What steps did you take? Did you have an action plan? How did you explain the approach to others? Did you collect data (from staff? From students?)? Did you consult stakeholders? Were there any problems in developing your approach? How were these resolved?

1. Who were the key stakeholders in the university that needed to be involved in developing the harm reduction approach?

What are the internal support pathways in the university for students with (problematic) drug use? How were these decided?

Which external stakeholders (outside the university) were involved? How does the policy interface with external support services/pathways?

How did you establish partnership between the key stakeholders?

Based on their experiences so far, would they say they had the right stakeholders involved (internal and external) or are their stakeholders they'd include with hindsight?

Were any stakeholders resistant to the harm reduction ethos? How were they persuaded that harm reduction would be the best course of action?

Were there any conflicts between the stakeholders regarding the harm reduction approach? How were these resolved?

1. Who drafted the harm reduction approach/policy? Were stakeholders consulted about the draft document? Did they provide feedback? What were the main comments/suggestions? How was alcohol incorporated into the approach/policy?
2. How was the new policy communicated internally within the university? Externally to local partners? Any problems encountered? How were these overcome?
3. What has been the response to the harm reduction approach/policy? Prompts: from university students, university staff, external partners or organisations etc. Were there any variations in the response of different stakeholders? Explain?
4. How committed do you think the university is to this type of approach/policy? Has it been sufficiently supported with resources and staffing? Why/why not?
5. Has the approach/policy been implemented? What has been done? What sort of interventions have been implemented? Any issues?
6. Were staff/students trained in implementing the new approach? How were the staff/students trained? Any problems with training? How were these resolved?
7. How are you monitoring the approach/policy? How are you measuring the impact? Have there been any changes to the policy as a result of this monitoring?
8. What do you see as the main barriers (internal and external) to implementing a harm reduction approach in universities? What do you see as the main facilitators (internal and external) to implementing a harm reduction approach in universities?
9. What have been the main achievements of your harm reduction approach/policy so far? Is there anything that needs to happen in the future to fully implement this approach? If yes, please describe.
10. What top tips would you give to other universities wanting to implement a harm reduction approach?

This set of questions will be asked for key informants from the universities that **have not** adopted a harm reduction approach.

1. Was your approach/policy towards substance use initiated by a specific person or group in the university? When was the issue first raised? What prompted this? Does the approach/policy include alcohol. If not, are there two separate policies/approaches (one for illegal drugs and one for alcohol)? Why?
2. How did you go about developing your existing substance use (alcohol / drug) approach/policy? When was the approach/policy (policies) last reviewed?

Prompts: What steps did you take? Did you have an action plan? How did you explain the approach to others? Did you collect data? Did you consult stakeholders? Were there any problems? How were these resolved?

1. Describe the ethos and principles underpinning your current substance use approach/policy? Why were these chosen as the underlying principles?
2. Was a harm reduction approach considered as an option?

What were the main reasons for not deciding on harm reduction?

1. Who was consulted on the type of approach to adopt? Internal stakeholders in the university? External stakeholders in the community? What was the main feedback from stakeholders?

Were there any conflicts between the different stakeholders regarding the type of approach to take? How were these resolved?

1. How was your substance use approach/policy communicated internally within the university? Externally to local partners? Any problems encountered? How were these overcome?
2. What has been the response to the approach/policy? Were there any variations in the response of different stakeholders? Explain?
3. How committed do you think the university is to this type of approach/policy? Has it been sufficiently supported with resources and staffing? Why/why not?
4. Has the approach/policy been implemented? What has been done? What sort of interventions have been implemented? Any issues?
5. Were staff/students trained in implementing the approach to substance use? How were the staff/students trained? Any problems with training? How were these resolved?
6. How are you monitoring the approach/policy? How are you measuring the impact? Have there been any changes to the approach/policy as a result of this monitoring?
7. What do you see as the main barriers (internal and external) to implementing a harm reduction approach in universities? What do you see as the main facilitators (internal and external) to implementing a harm reduction approach in universities?
8. What have been the main achievements of your substance use policy so far?
