# Supplementary material for: Building consensus for harm reduction approaches in UK universities: a qualitative study with staff and students
Source: Harm Reduct J. 2025 Dec 1;22:195. doi: 10.1186/s12954-025-01351-4 (PMC12667090; doi:10.1186/s12954-025-01351-4)
Supplement: Supplementary file 2 — Additional file 2. [file 12954_2025_1351_MOESM2_ESM.docx]

**Building consensus for harm reduction approaches in UK universities: a qualitative study with staff and students**

**Set of Questions/Prompts for Student Focus Groups**

**SECTION A: GROUP INFORMATION AND CONSENT [10 mins]**

**Hi, thanks for joining the group tonight. My name is [xxxx] from SOS-UK.**

**Before we start, we’ll tell you a bit more about the purpose of this focus group and run through some admin points.**

**We’re completing this focus group to find out about student perspectives on policies and action on alcohol and drugs within universities.**

**In terms of practicalities about this group…**

The information you provide will be used to develop a report produced by SOS-UK and Middlesex University.

We will treat everything you say in complete confidence. We will never identify you by name or any other information that could allow someone to recognise you.

Anonymous quotes from this group may be used in reports or other communications, however you will not be named.

The online group will automatically keep a record of everything you say, but this is just to help us with the analysis. Anything that personally identifies you will be removed from the record.

The record will not be viewed by anyone other than the staff at Middlesex University and SOS-UK working on this research.

You are free to withdraw from the research at any point, and you do not have to give a reason.

You can ask for your data to be withdrawn for up to two weeks following the focus group.

There is plenty to cover, but we’re really interested in hearing your views and experiences in detail so please answer the questions with as much detail as possible.

We’d also like you to be as open as you feel comfortable being - there are no right or wrong answers and we’re really interested in hearing your views and experiences in this area.

Please do not share anything from this group that might identify any of your fellow participants.

To thank you for your time, we'll send you £20 after the group – we’ll let you know at the end how to claim this.

This information has also been set out in the documents sent to you when you were invited to take part in the group.

**Are you happy to proceed with participating in the group today based on this information? Please put a thumbs up in the chat box to confirm.**

**SECTION B: UNIVERSITY POLICY AND ACTION [35 minutes]**

**First of all we’d like to know a bit about what you might have seen or heard about drugs or alcohol at your university.**

**What, if anything, are you aware of at your university linked to drugs or alcohol use?**

- *Who runs / leads this?*
- *Is it the university or students’ union?*
- *What is it trying to achieve?*
- *What are the main messages you took away from what you’ve seen or heard?*

**Still thinking about your university, what policies does it have related to drugs or alcohol?**

- How are these policies communicated to students?
- What are the main messages you have taken away from what you’ve seen of the policies?
- How would you describe the general aim of the policy, according to your understanding does it…
  - Try to prevent or stop students from taking drugs or drinking alcohol?
  - Or does it accept that students may take drugs or drink alcohol and try to reduce the harm that can be associated with these practices or behaviours?

**According to your understanding, what happens if a student is found in possession of drugs?**

Are they sent down (expelled), or suspended from university?

Are their parents informed?

Are police informed?

What else happens?

**How would you describe your university’s approach in general when a student is found in possession of drugs?**

Would you say it is supportive, or punitive?

What do you think of this approach?

What do you think should be done by your university when students are found in possession of drugs?

**Still according to your understanding, what happens if a student has problems related to their substance use at your university?**

What differences are there between problems with drug use compared to alcohol use?

**How would you describe your university’s approach in general when a student has problems related to drug use?**

Would you say it is supportive, or punitive?

**How would you describe the approach in relation to alcohol use?**

Would you say it is supportive, or punitive?

**What services or projects exist at your university for students who experience problems with drugs or alcohol?**

Do you know anything about…

Counselling services

Substance use services

Recovery groups

Drug testing services

Is there anything else you’re aware of?

Do you think these are easy to use/access?

What are the barriers to accessing them?

**What else should be done to support students who experience problems with alcohol or drugs?**

**SECTION C: UNDERSTANDING OF HARM REDUCTION [35 minutes]**

**Thanks for your input so far. Now we’d like to understand a bit more about your views on harm reduction.**

**Thinking about alcohol and drug use, what does the term ‘harm reduction’ mean to you?**

Is it different for alcohol and drugs?

**Thanks for that. Here’s a definition of harm reduction that we’ll use as the basis for the discussion from here on.**

“*Harm reduction refers to policies and practices that try to reduce the harm that people do to themselves or others from their drug use. It can be contrasted with primary prevention which tries to prevent people using drugs in the first place, or to stop them using once they’ve started*.” Drugwise, <https://www.drugwise.org.uk/harm-reduction-2/>

**Can you provide examples of harm reduction approaches to drugs and / or alcohol?**

Where have you come across these examples?

What were the main messages you can recall from these examples?

What kind of information were they providing?

Have you come across anything like this at your university?

According to your understanding, is this offered or supported by the university or the students’ union?

**In your view, is a harm reduction approach to drug use acceptable for universities to adopt?**

Can you tell me why you think it is acceptable?

If yes, are there any circumstances where harm reduction *would not* be an acceptable approach?

If no, are there any circumstances where harm reduction *would be* an acceptable approach?

**And how about alcohol consumption? Do you think it’s acceptable for universities to adopt a harm reduction approach to alcohol use?**

Can you tell me why you think it is acceptable?

Can you tell why you think it isn’t acceptable?

If yes, are there any circumstances where harm reduction *would not* be an acceptable approach?

If no, are there any circumstances where harm reduction *would be* an acceptable approach?

**How practical do you think it is for universities to adopt a harm reduction approach to drug use?**

Can you tell me why you think it isn’t practical?

Can you tell me more about why you think its practical?

**And how about alcohol consumption? Do you think it’s practical for universities to adopt a harm reduction approach to alcohol use?**

Can you tell me why you think it isn’t practical?

Can you tell me more about why you think its practical?

**Finally, is there anything else you’d like to share about your views of university action on drug and alcohol use?**

**SECTION D: GROUP CLOSE [5 mins]**

That’s all of my questions, is there anything you would like to add about the topics we’ve been speaking about that I’ve missed?

Or do you have any questions about the research?

Thank you for taking part in the group, your contributions have been really useful.

We’ll be in touch via email this week with a form to complete to allow us to send you the £20 you’ll receive as thanks for helping with our research. Please complete the form promptly to allow us to send the payment, which will be a direct payment to your bank.

If you have any questions about the payment, please get in touch at [email]

Thanks again for your time, you can close the browser to exit the group.
